# Plasma PZP level elevation: a potential correlate of diabetic kidney disease progression

**DOI:** 10.3389/fmed.2026.1833221

**Published:** 2026-05-28

**Authors:** Lin Mao, Ruili Yin, Longyan Yang, Dong Zhao

**Affiliations:** 1 Center for Endocrine Metabolism and Immune Diseases, Beijing Luhe Hospital, Capital Medical University, Beijing, China; 2 Beijing Key Laboratory of Artificial Intelligence and Cell-based Medical Engineering for Interdisciplinary Innovation and Clinical Translation, Beijing, China; 3 Laboratory for Clinical Medicine, Capital Medical University, Beijing, China

**Keywords:** diabetic kidney disease, hepatic cytokine, metabolic dysregulation, pregnancy zone protein, type 2 diabetes mellitus

## Abstract

**Background:**

Diabetic kidney disease (DKD) is the leading cause of chronic kidney disease (CKD) and end-stage renal disease in type 2 diabetes mellitus (T2DM) patients. Recent studies have demonstrated that pregnancy zone protein (PZP), a hepatic cytokine, plays a role in regulating glucose and lipid metabolism. This retrospective study aimed to investigate the connection between plasma PZP concentrations and DKD.

**Materials and methods:**

All participants were stratified by estimated glomerular filtration rate (eGFR) and urine albumin-to-creatinine ratio (UACR). The discovery cohort included 130 participants, from whom plasma samples were collected for four-dimensional data-independent acquisition (4D-DIA) quantitative proteomics. In contrast, plasma PZP concentrations were measured using the enzyme-linked immunosorbent assay (ELISA) in a validation cohort of 283 participants. Comparisons of clinical parameters, Spearman’s rho correlation analysis, logistic regression, and receiver operating characteristic (ROC) curve analysis were performed. Data were retrieved from public databases, and plasma PZP expression was simultaneously assessed in db/db mice and in primary hepatocytes treated with high glucose and palmitic acid (HGPA).

**Results:**

4D-DIA quantitative proteomics identified PZP as a differentially expressed protein in DKD patients. In the validation cohort, plasma PZP levels were significantly increased in DKD patients. Spearman’s rho correlation analysis revealed positive correlations between plasma PZP concentrations and systolic blood pressure (SBP), HbA1c, low-density lipoprotein (LDL), Cr, blood urea nitrogen (BUN), and UACR, as well as negative correlations with high-density lipoprotein (HDL) and eGFR. A multiple logistic regression analysis demonstrated a significant correlation between PZP and DKD progression (mild DKD: OR = 1.007; severe DKD: OR = 1.011). The ROC curve area was 0.8143. Plasma PZP levels in db/db mice were notably elevated, and a positive correlation with the UACR was observed. Moreover, PZP mRNA expression in HGPA-treated primary hepatocytes was twice as high as that in untreated cells.

**Conclusion:**

In summary, elevated plasma PZP levels are independently associated with DKD.

## Highlights

- This study reveals the relationship between higher plasma pregnancy zone protein (PZP) levels and the progression of diabetic kidney disease (DKD) in the Chinese population for the first time.
- Detecting plasma PZP concentrations in the early stage of DKD may provide a potential basis for timely intervention, thereby delaying or preventing disease progression.

## Introduction

In recent decades, the global prevalence of diabetes among adults has steadily increased. Among the various complications, diabetic kidney disease (DKD) is widely recognized as the most prevalent condition in patients with type 2 diabetes mellitus (T2DM) (1, 2). The incidence of DKD has surged sharply, with rates reaching up to 40% (3, 4). As a primary driver of chronic kidney disease (CKD) and end-stage renal disease (5), DKD develops as a result of abnormal glucose and lipid metabolism. This metabolic dysfunction triggers a gradual deterioration in renal function, commonly culminating into the need for renal replacement therapy in advanced stages. The consequent decline in patients’ quality of life and the growing socioeconomic burden highlight the urgent need for more effective prevention and treatment strategies for DKD (4, 6). In recent medical research, the significance of inter-organ crosstalk has gained increasing attention. The liver, as the body’s largest metabolic organ, secretes hepatic cytokines that regulate the functions of other organs via multiple signaling pathways. Previous research has indicated that in patients diagnosed with non-alcoholic fatty liver disease (NAFLD), greater severity of liver fibrosis is linked to a marked rise in the incidence of DKD. In addition, hepatic cytokines have been confirmed to mediate the pathological progression of DKD (7–9). Against this backdrop, our team investigated the pathogenesis and underlying mechanisms of DKD by focusing on liver–kidney interactions (10, 11).

Pregnancy zone protein (PZP) is a highly conserved 360 kDa glycoprotein that belongs to the *α*-macroglobulin family (12). It binds to high-affinity receptors located on macrophage surfaces, thereby exerting immunosuppressive effects (12, 13). Moreover, the structural composition of PZP contains multiple protease cleavage sites, endowing it with potential protease inhibitory activity (14, 15). As a protein with strong liver-specific expression, PZP can be detected in various bodily fluids (16). Over the past few years, its role in metabolic processes has gradually come to light. A study focusing on PZP during pregnancy indicated that plasma PZP levels are associated with diabetes mellitus (DM) and DM complicated by hypertensive disorders of pregnancy (17, 18). Furthermore, PZP participates in lipid metabolism regulation and was significantly modulated 6 months after the weight maintenance phase in a large multicenter dietary trial, supporting its potential role as a novel biomarker for sustained body weight management (19, 20). In studies, obese mice exhibited notably lower PZP concentrations in both their liver and plasma compared to the control group. In addition, intermittent fasting (IF) followed by refeeding led to increased plasma PZP concentrations in both obese mice and human patients. IF combined with PZP overexpression improved insulin sensitivity and enhanced the capacity to clear exogenous glucose (20, 21). Under IF conditions, PZP promoted heat production in brown adipose tissue (BAT) and enhanced energy consumption through uncoupling protein 1 (UCP1) (21). Recent research has demonstrated that PZP contributes to age-related CKD progression, primarily through the regulation of CD8 + central memory T cells (22). Drawing on the available evidence, we hypothesized that PZP may be linked to DKD; however, the nature of this association and its potential functional role require further clarification. The present study aimed to evaluate the connection between plasma PZP levels and DKD development and progression.

## Materials and methods

### Participants

This study included DKD patients and T2DM patients from the National Metabolic Management Center (MMC) of Beijing Luhe Hospital, affiliated with Capital Medical University, from 2019 to 2023, along with healthy individuals undergoing routine examinations in the physical examination department. The diagnostic criteria for DKD were based on the clinical practice guidelines of the Kidney Disease Outcomes Quality Initiative (KDOQI), whereas the T2DM diagnosis followed the 1999 criteria developed by the World Health Organization (WHO). The exclusion criteria included type 1 diabetes, ketoacidosis, hypertonic state, acute severe infection, cancer, and severe cardiovascular disease.

A total of 413 participants were ultimately included in this study. Among them, 104 participants (24 healthy participants and 80 DKD patients) underwent four-dimensional data-independent acquisition (4D-DIA) quantitative proteomics to screen for differentially expressed genes. Healthy participants were required to meet the following criteria: Fasting blood glucose (FBG) ≤ 6.1 mmol/L, urine albumin-to-creatinine ratio (UACR) < 30 mg/g, and estimated glomerular filtration rate (eGFR) ≥ 90 mL/min/1.73m^2^. For DKD patients, the inclusion criteria were FBG > 7.0 mmol/L, UACR≥100 mg/g, or eGFR≤60 mL/min/1.73m^2^.

In addition, the remaining 283 participants were divided into four groups based on the eGFR and UACR and UACR: 53 healthy participants, 77 T2DM participants, 92 mild DKD participants, and 61 severe DKD participants. Healthy participants were defined as described previously. T2DM patients had FBG > 7.0 mmol/L, eGFR ≥ 90 mL/min/1.73m^2^, and UACR < 30 mg/g. Mild DKD patients had FBG > 7.0 mmol/L, eGFR ≥ 90 mL/min/1.73m^2^, and UACR 30–300 mg/g. Severe DKD patients met the following criteria: FBG > 7.0 mmol/L, eGFR < 60 mL/min/1.73m^2^, and UACR > 300 mg/g. The enzyme-linked immunosorbent assay (ELISA) was used to measure plasma PZP levels in these four groups, and further analysis of data was conducted. A diagnosis of hypertension was defined as having a blood pressure value of ≥140/90 mmHg or the use of any antihypertensive medication. Body mass index (BMI) was calculated by dividing weight (in kilograms) by the square of height (in meters), which is expressed as kg/m^2^.

### Blood collection and biochemical and anthropometric measurements

All clinical information of the participants was collected and recorded from the electronic clinical management record system of Beijing Luhe Hospital. After 8–12 h of overnight fasting, venous blood samples were drawn from each participant. Specialized instruments were used to measure the following indicators: fasting blood glucose (FBG), glycated hemoglobin (HbA1c), triglycerides (TG), total cholesterol (TC), high-density lipoprotein cholesterol (HDL-C), low-density lipoprotein cholesterol (LDL-C), serum creatinine (Cr), uric acid (UA), blood urea nitrogen (BUN), and UACR. For Chinese individuals, the eGFR (mL/min/1.73m^2^) was calculated using the following formula: 186*(Crea*0.011)^−1.154^ *(age)^−0.203^*(0.742 if female/1 if male)*1.233.

### Proteomic analysis

Plasma samples from the control and DKD groups were subjected to 4D-DIA quantitative proteomics by Shanghai BioTree Biotechnology Co., Ltd. (Shanghai, China). The raw data pre-processing involved two key steps: (1) proteins with at least one unique peptide segment were retained, and (2) missing values were imputed using the half-minimum value method. Differentially expressed proteins were screened using Student’s *t*-test under the following conditions: |Log_2_^Fold change^| > 1 and p.adj < 0.05.

### Gene expression data analysis

Public data from the National Center for Biotechnology Information (NCBI) were analyzed to evaluate the expression of two differentially expressed genes across various tissues: SRY-box transcription factor 30 (SOX30, gene ID: 11063) and PZP (gene ID: 5858). The expression level of PZP in various hepatic cell types was obtained from the Tabula Muris single-cell database.

### Measurement of PZP and the UACR

Each participant who met the inclusion criteria had approximately 2–3 mL of venous blood drawn after fasting for 8–12 h. According to the operating manual, human and mouse plasma PZP levels (ng/mL) were measured using ELISA kits (USEG324Hu and USG324Mu, Cloud Clone Corp., formerly Uscn Life Science, Inc.). The intra-assay coefficients of variation for PZP were less than 10%, while the inter-assay coefficients of variation were less than 12%.

Urine samples were collected from 12-week-old db/m and db/db mice. Urinary microalbumin levels were quantitatively detected using a mouse MAU ELISA Kit (Elabscience, E-EL-M0792). Urinary creatinine was measured using a creatinine assay kit (Nanjing Jiancheng, C011-2-1). The UACR was calculated by taking the ratio of urinary microalbumin to urinary creatinine.

### Isolation of primary mouse hepatocytes and quantitative real-time PCR

Primary mouse hepatocytes were isolated from 8-week-old male C57BL/6J mice using the collagenase digestion method (23). After treatment with high glucose and palmitic acid (HGPA) (HG, A2494001; PA, P9767, Sigma-Aldrich) for 24 h at a density of 70–80%, cDNA was extracted from the cells under different treatment conditions using the TRIzol method, and RT-qPCR was performed. PZP expression levels were quantified using the 2^^(-ΔΔCt)^ method. The primer sequences are presented in the table below:

**Table:** 

Primer name	Sequence (5′ to 3′)
Mouse-PZP-forward primer Mouse-PZP-reverse primer β-actin-forward primer β-actin-reverse primer	GGTCCCGTCAGAGGTCTATTCGTCTGTGAGGAGCTTCGTTTG TCATGAAGTGTGACGTGGACATC CAGGAGGAGCAATGATCTTGATCT

### Statistical analysis

This study employed R software (version 4.1.2) for statistical analysis and graphical visualization. Continuous variables with a normal distribution were presented as mean ± standard deviation, and significance was tested using Student’s *t*-test. Skewed variables were displayed as median (interquartile range: 25 to 75%) and analyzed using the Mann–Whitney *U*-test. Categorical variables were expressed as proportions and compared using the chi-squared test. Spearman’s rho correlation was used to evaluate the correlation between PZP and other variables. Following the adjustment for known risk factors and variables with baseline statistical differences, a multiple logistic regression analysis was performed to explore the independent correlation between PZP and DKD. The ability of PZP to predict the occurrence and progression of DKD was evaluated using receiver operating characteristic (ROC) curve analysis with corresponding 95% confidence intervals. Differences were deemed statistically significant when the *p*-value was <0.05.

## Results

### Identification and functional classification of PZP

**Figure 1:**
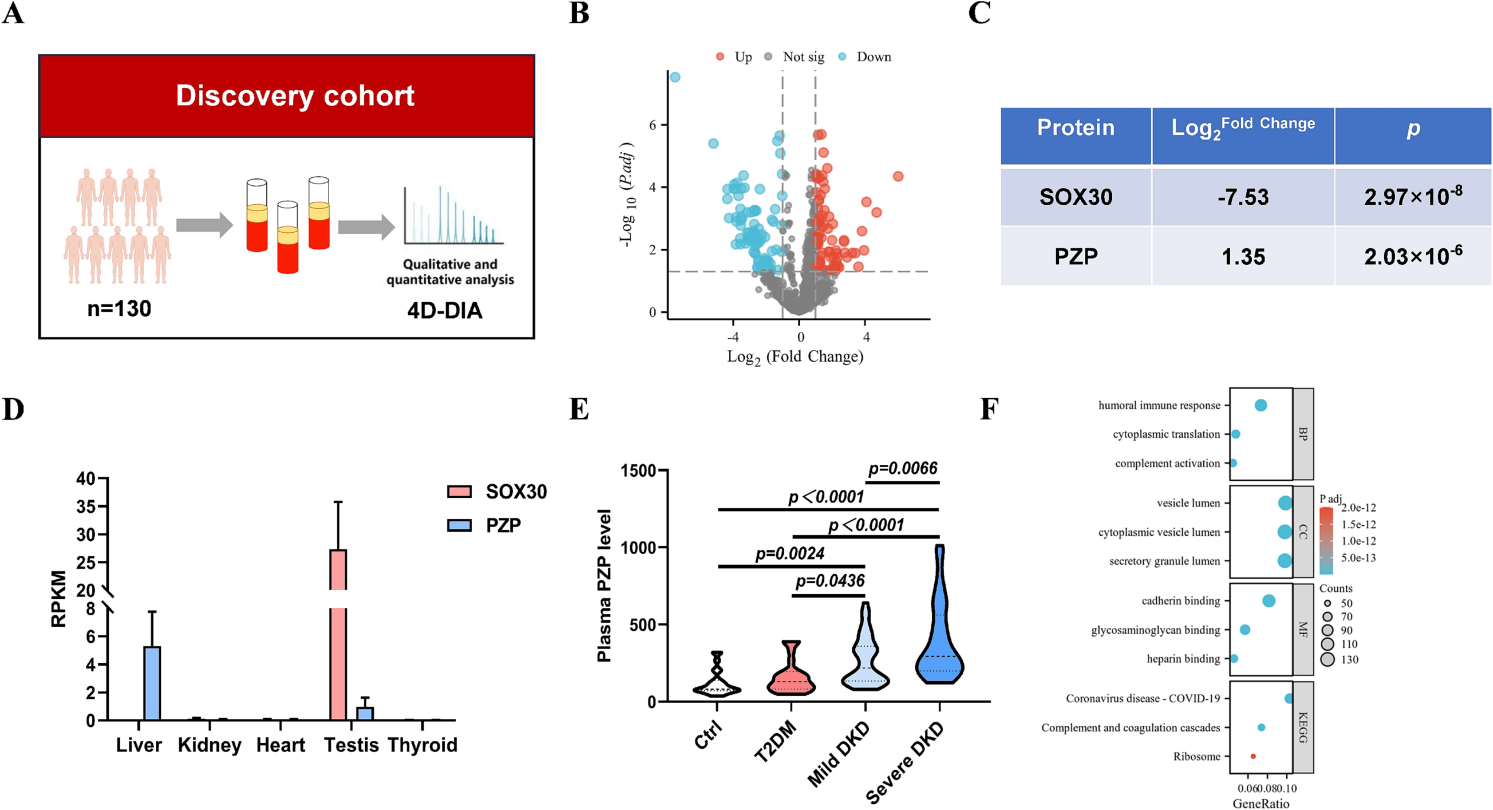
Identification and functional classification of DEPs. **(A)** The flowchart of the discovery cohort; **(B)** differentially expressed proteins in the control and DKD groups; **(C)** top two proteins ranked by *p*-value; **(D)** expression levels of SOX30 and PZP in different organizations from the NCBI; **(E)** plasma PZP levels in the discovery cohort; **(F)** GO and KEGG enrichment bubble plots of differentially expressed proteins screened based on plasma PZP levels. SOX30, SRY-box transcription factor 30; PZP, pregnancy zone protein; T2DM, type 2 diabetes mellitus. Visual graphs in this study were generated with R software (version 4.1.2).

To explore the pathological mechanisms driving the onset and progression of DKD, we collected plasma samples from DKD patients and control participants for 4D-DIA proteomic analysis. The discovery cohort ultimately consisted of 130 participants, who were divided into four groups based on the eGFR and UACR: 24 healthy participants, 26 T2DM patients, 51 mild DKD patients, and 29 severe DKD patients (Figure 1A). General demographic and clinical data of the cohort are presented in Table 1.

**Table 1:** Comparison of clinical characteristics of the study population stratified by DKD severity in the discovery cohort.

Characteristic	Control	T2DM	Mild DKD	Severe DKD	*p*
Sex (M/F)	24 (9/15)	26 (13/13)	51 (24/27)	29 (11/18)	0.701
Age (years)	54.6 ± 7.6	69.5 ± 3.6	63.7 ± 7.5	65.9 ± 4.4	<0.0001*
BMI (kg/m^2^)	24.1 ± 3.1	24.8 ± 2.3	24.9 ± 2.2	24.7 ± 2.1	0.633
SBP (mmHg)	115.2 ± 13.9	134.0 ± 14.3	133.9 ± 15.8	137.1 ± 18.4	<0.0001*
DBP (mmHg)	74.8 ± 11.6	74.6 ± 9.7	75.9 ± 10.0	79.4 ± 11.2	0.308
FBG (mmol/L)	4.8 (4.6,5.3)	7.6 (6.4,10.1)	7.9 (6.8,9.9)	8.9 (7.6,10.4)	<0.0001*
HbA1c (%)	5.7 ± 0.4	7.4 ± 1.2	8.0 ± 1.5	9.2 ± 2.1.	<0.0001*
TG (mmol/L)	1.2 (0.9,1.8)	0.9 (0.7,1.4)	1.7 (1.3,2.2)	1.8 (1.1,2.6)	<0.0001*
TC (mmol/L)	5.5 ± 0.9	4.3 ± 1.0	4.9 ± 1.2	4.8 ± 1.5	0.004*
HDL (mmol/L)	1.5 ± 0.4	1.4 ± 0.3	1.2 ± 0.3	1.1 ± 0.2	<0.0001*
LDL (mmol/L)	3.2 ± 0.7	2.5 ± 0.7	3.1 ± 0.9	3.0 ± 1.1	0.012*
eGFR, (mL/min/1.73m^2^)	92.8 ± 12.8	89.1 ± 10.9	77.3 ± 26.7	44.5 ± 11.7	<0.0001*
UACR (mg/g)	6.04 ± 4.7	8.8 ± 6.3	223.7 ± 349.5	937.3 ± 931.1	<0.0001*
UA (μmol/L)	370.1 ± 142.9	281.1 ± 85.3	363.8 ± 87.4	377.1 ± 78.8	0.001*
Cr (μmol/L)	74.4 ± 13.0	70.2 ± 15.1	88.4 ± 27.6	143.7 ± 79.4	<0.0001*
BUN (mmol/L)	5.3 (4.6,6.4)	5.2 (4.5,6.1)	6.0 (5.3,7.9)	8.6 (7.1,10.4)	<0.0001*
PZP (ng/mL)	121.1 ± 81.5	162.6 ± 106.3	266.9 ± 154.5	392.2 ± 247.3	<0.0001*

DKD, diabetic kidney disease; BMI, body mass index; SBP, systolic blood pressure; DBP, diastolic blood pressure; FBG, fasting blood glucose; HbA1c, glycated hemoglobin; TG, triglycerides; TC, total cholesterol; HDL, high-density lipoprotein; LDL, low-density lipoprotein; eGFR, estimated glomerular filtration rate; UACR, urine albumin-to-creatinine ratio; UA, uric acid; Cr, creatinine; BUN, blood urea nitrogen; PZP, pregnancy zone protein. *p < 0.05. Bold values in indicates statistically significant differences (*p* < 0.05).

As illustrated in the volcano plot, using |Log_2_^Fold Change^| > 1 and p.adj < 0.05 as screening criteria, 186 differentially expressed proteins were identified between 24 healthy participants and 80 DKD patients, including 88 upregulated proteins and 98 downregulated proteins (Figure 1B). Ranked by *p*-value in ascending order, the top two differentially expressed genes were SOX30 and PZP (Figure 1C). Data retrieved from the NCBI database showed that only PZP exhibited specifically high expression in the liver (Figure 1D). The results from 4D-DIA quantitative proteomics showed that plasma PZP levels progressively increased with DKD progression (Figure 1E). Specifically, plasma PZP levels in mild and severe DKD patients were 1.9-fold and 2.8-fold higher than those in healthy individuals, respectively (*p* < 0.0001). Among the four groups, lipid levels displayed a trend of initial decrease followed by an increase, while no significant differences were observed in sex, BMI, or systolic blood pressure (SBP).

To explore potential signaling pathways through which PZP contributes to DKD, participants were divided into high and low PZP expression subtypes based on the median PZP expression level. Gene Ontology (GO) and Kyoto Encyclopedia of Genes and Genomes (KEGG) enrichment analyses were then performed on differentially expressed genes between the two subtypes. GO enrichment analysis showed that biological processes were mainly enriched in human immune response, cytoplasmic translation, and complement activation; cellular components were mainly enriched in vesicle lumen, cytoplasmic vesicle lumen, and secretory granule lumen; and molecular functions were mainly enriched in cadherin binding, glycosaminoglycan binding, and heparin binding. KEGG pathway analysis indicated significant enrichment in coronavirus disease–COVID–19, complement and coagulation cascades, and ribosome pathways (Figure 1F). These enrichment results suggest that PZP may be involved in DKD progression through immune- and complement-related pathways.

### Plasma PZP levels were upregulated and correlated with the UACR

**Figure 2:**
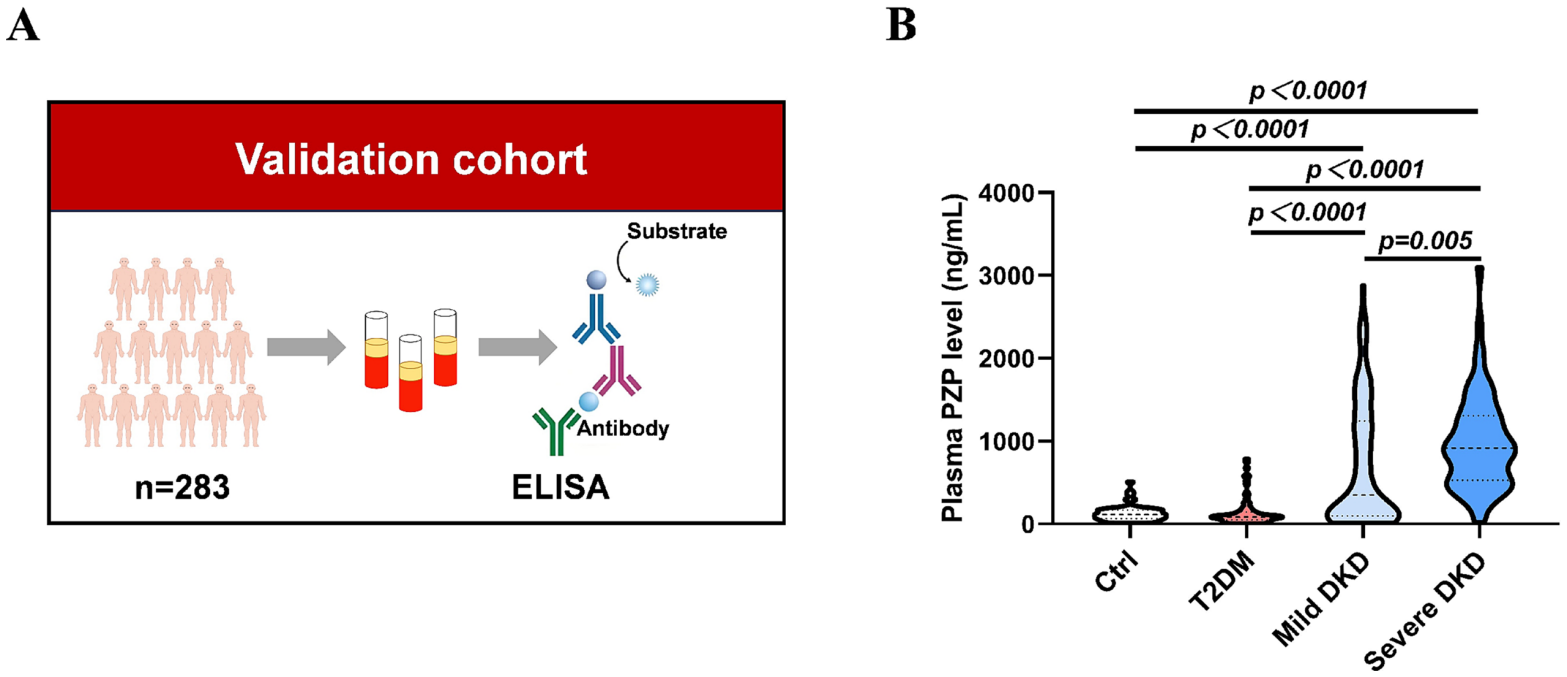
Plasma PZP levels in the validation cohort. **(A)** The flowchart of the validation cohort; **(B)** plasma PZP levels of participants in different groups. PZP, pregnancy zone protein; T2DM, type 2 diabetes mellitus.

To further validate the above findings, we expanded the sample size by establishing an ELISA-based validation cohort. The validation cohort eventually included 283 participants, who were divided into 53 healthy participants, 77 T2DM patients, 92 mild DKD patients, and 61 severe DKD patients (Figure 2A). General information about the validation cohort is provided in Table 2. The mean ages were 43.4 ± 11.5, 61.2 ± 9.9, 50.8 ± 11.1, and 61.3 ± 8.7, respectively (*p* < 0.0001). Plasma PZP levels in patients with mild and severe DKD were 5.1-fold and 7.1-fold higher than those in healthy individuals, respectively (*p* < 0.0001) (Figure 2B). The correlation between plasma PZP levels and clinical variables was assessed using Spearman’s rho correlation (Table 3). Without adjustment for confounding factors, plasma PZP levels were positively correlated with SBP (*r* = 0.144, *p* = 0.02), FBG (*r* = 0.119, *p* = 0.045), HbA1c (*r* = 0.315, *p* < 0.0001), BMI (*r* = 0.186, *p* = 0.003), TG (*r* = 0.236, *p* < 0.0001), Cr (*r* = 0.294, *p* < 0.0001), UA (*r* = 0.185, *p* = 0.002), UACR grade (*r* = 0.17, *p* = 0.004), and BUN (*r* = 0.118, *p* = 0.049) and negatively correlated with sex (*r* = −0.154, *p* = 0.009), HDL (*r* = −0.34, *p* < 0.0001), and eGFR (*r* = −0.243, *p* < 0.0001). Partial correlation analysis adjusted for age and sex showed that plasma PZP levels were positively correlated with FBG (*r* = 0.216, *p* = 0.002), HbA1c (*r* = 0.359, *p* < 0.0001), TG (*r* = 0.206, *p* = 0.004), and Cr (*r* = 0.232, *p* = 0.001) and negatively correlated with HDL (*r* = −0.270, *p* < 0.0001) and eGFR (*r* = −0.187, *p* = 0.008).

**Table 2:** Comparison of clinical characteristics of the study population stratified by DKD severity in the validation cohort.

Characteristic	Control	T2DM	Mild DKD	Severe DKD	*p*
Sex (M/F)	53 (12/41)	77 (41/36)	92 (45/47)	61 (48/13)	<0.0001*
Age (years)	43.4 ± 11.5	61.2 ± 9.9	50.8 ± 11.1	61.3 ± 8.7	<0.0001*
BMI (kg/m^2^)	23.4 ± 3.1	25.9 ± 3.4	26.7 ± 3.9	26.6 ± 3.0	<0.0001*
SBP (mmHg)	118.6 ± 19.6	132.6 ± 17.8	131.7 ± 17.6	137.0 ± 17.0	<0.0001*
DBP (mmHg)	71.7 ± 14.6	79.3 ± 11.6	79.1 ± 11.1	78.9 ± 12.6	0.002*
FBG (mmol/L)	4.9 (4.8,5.3)	7.7 (6.7,8.6)	8.3 (6.4,13.7)	7.3 (6.3,9.3)	<0.0001*
HbA1c (%)	5.6 ± 0.2	7.8 ± 1.9	9.6 ± 2.4	8.1 ± 2.0	<0.0001*
TG (mmol/L)	0.8 (0.6,1.1)	1.3 (0.9,2.3)	1.5 (1.0,2.2)	1.4 (1.2,2.5)	<0.0001*
TC (mmol/L)	4.7 ± 0.7	4.8 ± 1.1	5.1 ± 1.6	4.8 ± 1.4	0.282
HDL (mmol/L)	1.6 ± 0.3	1.3 ± 0.3	1.2 ± 0.3	1.1 ± 0.3	<0.0001*
LDL (mmol/L)	2.9 ± 0.6	2.9 ± 1.0	3.1 ± 1.0	3.1 ± 1.1	0.213
eGFR (mL/min/1.73m^2^)	101.7 ± 18.6	94.3 ± 11.7	102.3 ± 15.7	53.5 ± 5.8	<0.0001*
Grade of UACR					<0.0001*
Mild (*n*)	45	77	57	24	
Moderate (*n*)	5	0	18	19	
Severe (*n*)	3	0	17	18	
UA (μmol/L)	287.2 ± 78.2	317.2 ± 76.7	326.6 ± 96.6	373.0 ± 95.7	<0.0001*
Cr (μmol/L)	67.6 ± 12.2	64.5 ± 10.7	63.4 ± 12.1	102.3 ± 12.7	<0.0001*
BUN (mmol/L)	4.7 (4.0,5.5)	5.4 (4.5,6.2)	4.4 (3.8,5.8)	6.7 (5.7,7.5)	<0.0001*
PZP (ng/mL)	129.3 ± 97.1	143.9 ± 172.8	695.1 ± 740.7	976.9 ± 571.2	<0.0001*

DKD, diabetic kidney disease; BMI, body mass index; SBP, systolic blood pressure; DBP, diastolic blood pressure; FBG, fasting blood glucose; HbA1c, glycated hemoglobin; TG, triglycerides; TC, total cholesterol; HDL, high-density lipoprotein; LDL, low-density lipoprotein; eGFR, estimated glomerular filtration rate; UACR, urine albumin-to-creatinine ratio; UA, uric acid; Cr, creatinine; BUN, blood urea nitrogen; PZP, pregnancy zone protein. *p < 0.05. Bold values in indicates statistically significant differences (*p* < 0.05).

**Table 3:** Correlation analysis between PZP and other variables.

Variables	Plasma PZP		Plasma PZP*	
	*r*	*p*	*r*	*p*
Sex	**-0.154**	0.009*		
Age (years)	0.028	0.641		
SBP (mmHg)	**0.144**	0.020*	0.067	0.35
DBP (mmHg)	0.078	0.21	−0.044	0.539
FBG (mmol/L)	**0.119**	0.045*	**0.216**	0.002*
HbA1C (%)	**0.315**	<0.0001*	**0.359**	<0.0001*
BMI (kg/m^2^)	**0.186**	0.003*	0.109	0.128
TG (mmol/L)	**0.236**	<0.0001*	**0.206**	0.004*
TC (mmol/L)	0.019	0.745	0.096	0.177
HDL (mmol/L)	**-0.34**	<0.0001*	**−0.27**	<0.0001*
LDL (mmol/L)	0.083	0.164	0.125	0.079
eGFR (mL/min/1.73m^2^)	**-0.243**	<0.0001*	**−0.187**	0.008*
Cr (μmol/L)	**0.294**	<0.0001*	**0.232**	0.001*
UA (μmol/L)	**0.185**	0.002*	0.091	0.201
Grade of UACR	**0.17**	0.004*	0.107	0.073
BUN (mmol/L)	**0.118**	0.049*	0.091	0.201

*p*-values were obtained using Spearman’s correlation analysis. *Partial correlation analysis was adjusted for age and sex. *p < 0.05 was regarded as significant.

SBP, systolic blood pressure; DBP, diastolic blood pressure; FBG, fasting blood glucose; HbA1c, glycated hemoglobin; BMI, body mass index; TG, triglycerides; TC, total cholesterol; HDL, high-density lipoprotein; LDL, low-density lipoprotein; eGFR, estimated glomerular filtration rate; Cr, creatinine; UA, uric acid; UACR, urine albumin-to-creatinine ratio; BUN, blood urea nitrogen. Bold values in indicates statistically significant differences (*p* < 0.05).

### PZP levels increased in db/db mice and HGPA-treated primary hepatocytes

**Figure 3:**
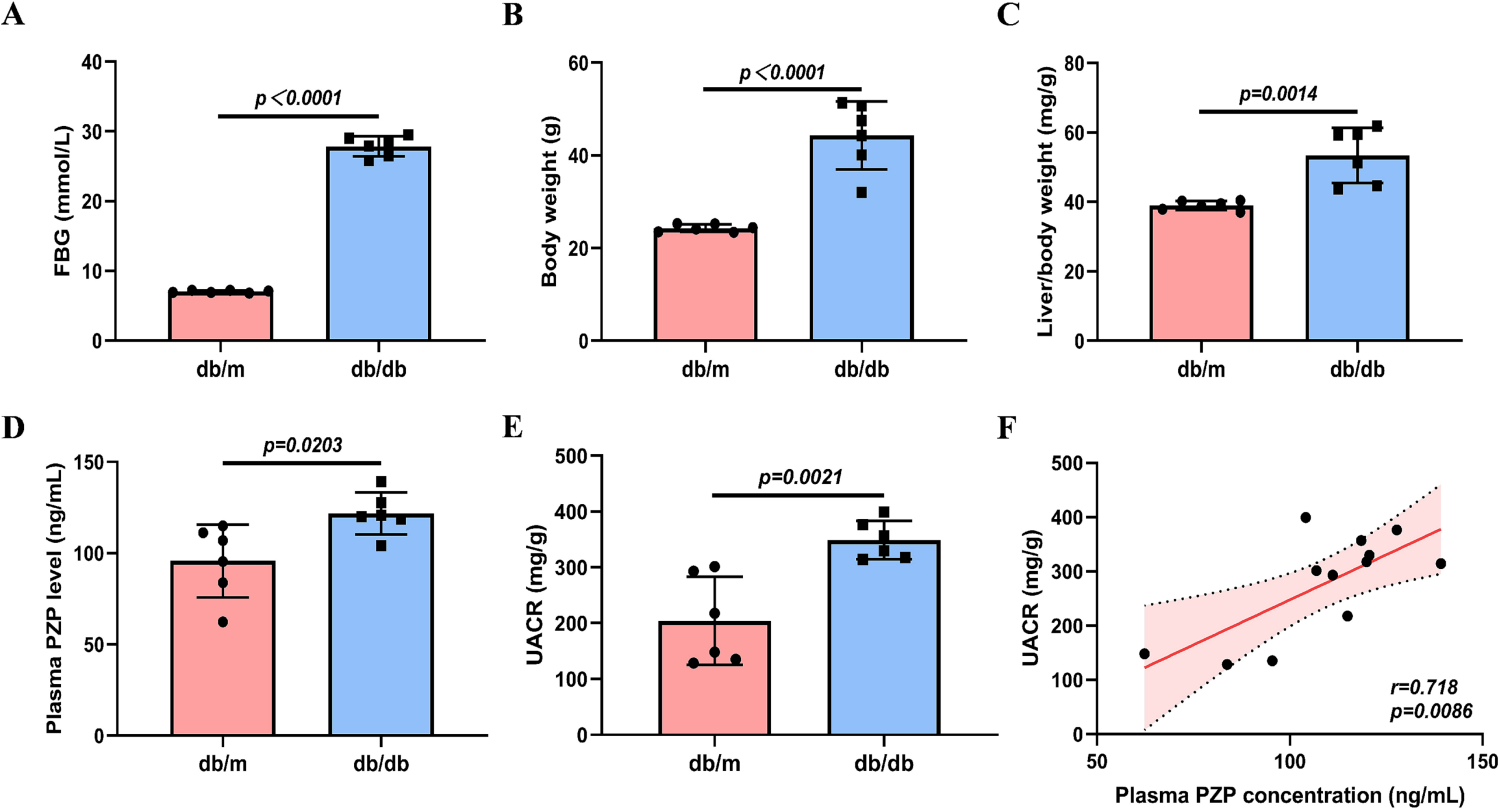
PZP expression in the T2DM animal model. **(A)** Fasting blood glucose in db/m and db/db mice; **(B)** body weight of db/m and db/db mice; **(C)** ratio of liver weight to body weight in db/m and db/db mice; **(D)** plasma PZP levels in db/m and db/db mice; **(E)** UACR in db/m and db/db mice; **(F)** correlation between plasma PZP concentrations and the UACR in the T2DM animal model.

**Figure 4:**
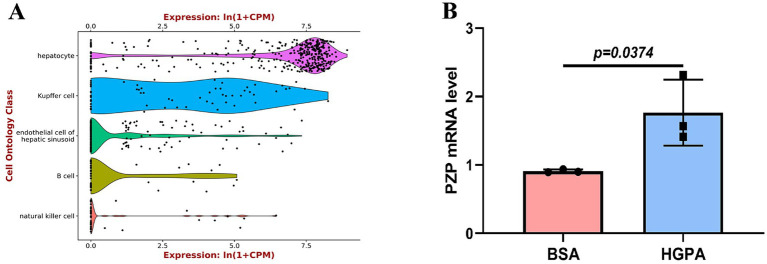
PZP expression levels in public databases and primary hepatocytes. **(A)** PZP expression in the liver from the Tabula Muris database; **(B)** PZP expression levels in primary hepatocytes treated with HGPA. PZP, pregnancy zone protein; HGPA, high glucose and palmitic acid. Visual graphs in this study were generated with R software (version 4.1.2).

To further clarify the role of PZP in DKD, we used db/db mice (a classic murine DKD model). Compared to db/m mice, db/db mice showed significantly increased FBG, body weight, and liver-to-body weight ratio (Figures 3A–C). The UACR showed a significant increase in db/db mice compared to db/m mice (204.1 ± 79.1 vs. 349.1 ± 34.4 mg/g, respectively) (Figure 3D). Compared to db/m mice, plasma PZP levels in db/db mice were significantly higher and positively correlated with the UACR (*r* = 0.718, *p* = 0.0086) (Figures 3E,F). By searching the Tabula Muris single-cell database, PZP was found to be highly expressed in hepatocytes (Figure 4A). RT-qPCR results showed that PZP mRNA levels were significantly elevated in primary hepatocytes treated with HGPA (Figure 4B).

### Correlation between plasma PZP levels and the progression of DKD

Plasma PZP concentrations gradually increased with DKD progression in both the discovery cohort and the validation cohort. Multiple logistic regression further analyzed the interrelation between plasma PZP levels and DKD progression in the validation cohort using different models (Table 4). After adjusting for confounding factors including age, sex, SBP, FBG, LDL, HDL, BUN, and Cr, plasma PZP levels remained significantly correlated with DKD progression (mild DKD: OR = 1.007, 95% CI: 1.003–1.011, *p* = 0.001; severe DKD: OR = 1.011, 95% CI: 1.005–1.018, *p* < 0.0001).

**Table 4:** Independent effect of plasma PZP on the development of DKD.

	PZP (ng/mL)	
	OR (95% CI)	*p*
Model 1		
Control	Ref.	
T2DM	1.000 (0.998-1.003)	0.814
Mild DKD	1.005 (1.002-1.007)	<0.0001*
Severe DKD	1.006 (1.003-1.008)	<0.0001*
Model 2		
Control	Ref.	
T2DM	1.003 (0.999-1.007)	0.18
Mild DKD	1.006 (1.002-1.010)	0.002*
Severe DKD	1.009 (1.004-1.014)	<0.0001*
Model 3		
Control	Ref.	
T2DM	1.004 (1.000-1.009)	0.071
Mild DKD	1.007 (1.003-1.011)	0.001*
Severe DKD	1.011 (1.005-1.018)	<0.0001*

Model 1: Adjusted for age and sex.

Model 2: Adjusted for SBP, Cr, and BUN plus the covariates in model 1.

Model 3: Adjusted for LDL, HDL, and FBG plus the covariates in model 2.

PZP, pregnancy zone protein; T2DM, type 2 diabetes mellitus. *p-value < 0.05. Bold values in indicates statistically significant differences (*p* < 0.05).

### ROC analysis for diagnosing DKD

**Figure 5:**
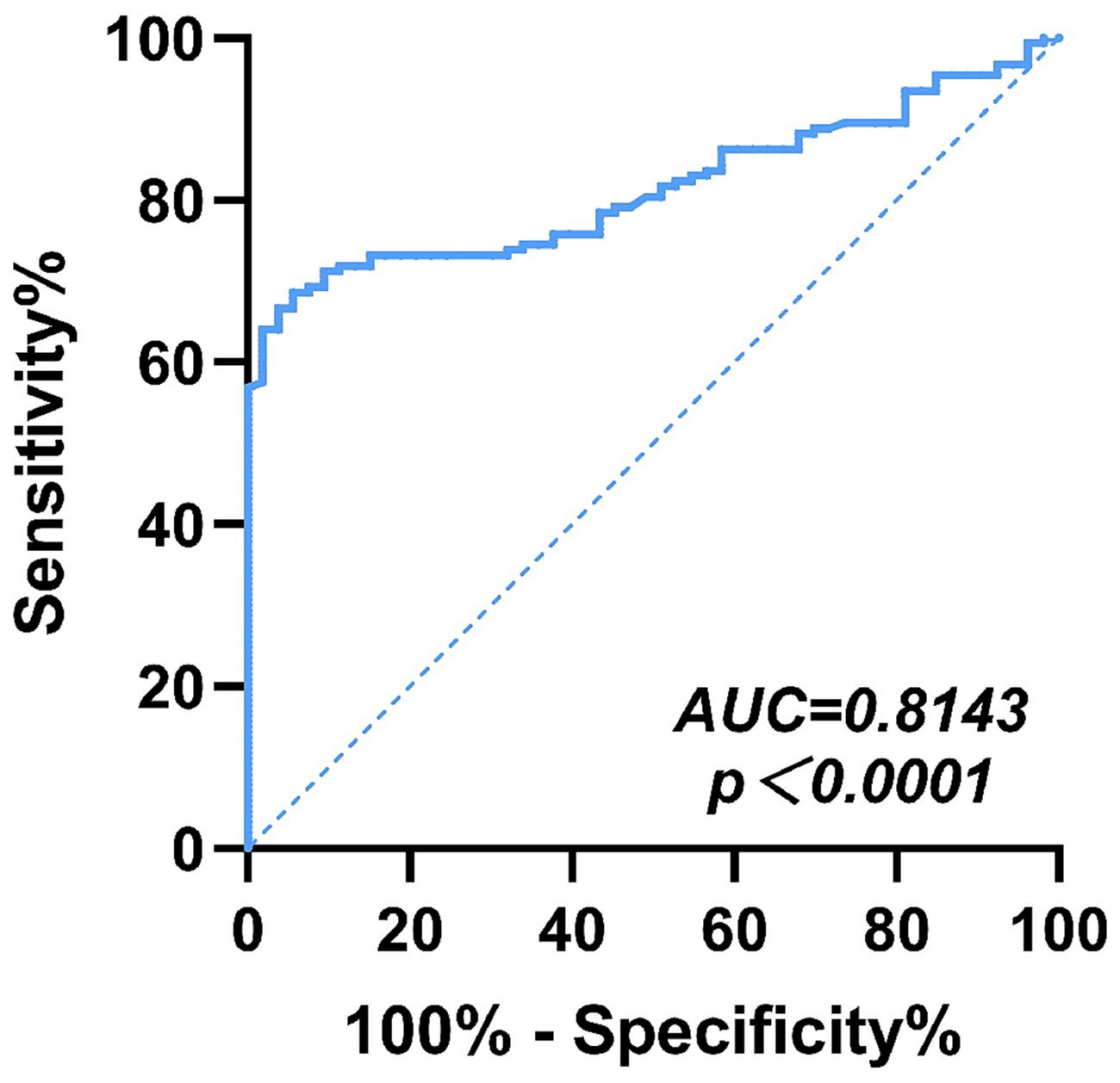
ROC curve analysis of plasma PZP levels for DKD in the validation cohort. ROC curve analysis was performed to estimate the predictive ability of PZP to distinguish DKD.

The diagnostic ability of PZP for DKD was evaluated using ROC curve analysis. The results showed that the area under the ROC curve was 0.8143 (95% CI: 0.7584–0.8701, *p* < 0.0001). At a plasma PZP cutoff of 379.2 ng/mL, the sensitivity and specificity for diagnosing DKD were 61.4 and 93.1%, respectively (Figure 5).

## Discussion

DKD ranks among the most severe chronic complications of DM, and its high mortality and disability rates impose a substantial economic burden on society. However, the current understanding of the underlying pathological mechanisms of DKD remains limited. By integrating clinical data, public transcriptomic databases, and animal/cell models, this study preliminarily observed that PZP expression was elevated in the plasma of both DKD patients and db/db mice. PZP levels are closely correlated with the initiation and progression of DKD—suggesting a potential functional role of PZP in DKD development.

PZP, a homodimeric glycoprotein cross-linked by disulfide bonds, is primarily secreted by the liver. Early research primarily focused on its physiological role during pregnancy; however, PZP has recently been shown to regulate key signaling pathways involved in energy metabolism, insulin sensitivity, and inflammatory responses. Meanwhile, PZP has been implicated in the pathogenesis of obesity-related disorders, metabolic diseases, chronic inflammatory diseases, and malignancies. Despite this, its specific function in DKD remains unclear and requires further investigation.

PZP is involved in both lipid and glucose metabolism, two core pathways underlying DKD pathogenesis. Previous studies have reported the critical role of PZP in lipid metabolism. Cell surface glucose-regulated protein 78 (GRP78) interacts with circulating PZP, which triggers the activation of the p38 mitogen-activated protein kinase-activating transcription factor 2 (p38 MAPK-ATF2) signaling pathway in BAT, thereby upregulating UCP1 expression to promote thermogenesis. PZP-knockout mice are more susceptible to high-fat diet-induced obesity, while recombinant PZP administration or adipose tissue-specific PZP overexpression confers resistance to obesity and reduces lipid accumulation (20, 21, 24). As a ligand for low-density lipoprotein receptor-related protein (LRP, a member of the low-density lipoprotein receptor family), PZP plays a pivotal role in regulating lipoprotein metabolism (25). Additional studies have shown that proteolytic processing can sequester the binding interface between LRP1 and PZP. Notably, LRP1 protects lysosomal integrity, mitigates autophagic dysfunction, and alleviates oxidative imbalance in DKD (26)—suggesting that PZP may exert a protective effect in DKD by regulating lipid metabolism.

Several studies have indicated that PZP may also be involved in the regulation of glucose metabolism. A pregnancy-related clinical study reported lower plasma PZP concentrations in patients with DM and in those with DM complicated by gestational hypertension compared to healthy participants (18). Adipose tissue-specific PZP overexpression significantly improves glucose clearance and insulin sensitivity in mice, likely by promoting angiogenesis and the expression of genes related to glucose utilization, thereby enhancing white adipose tissue metabolic activity. Further research is warranted to clarify the specific role and underlying mechanisms of PZP in glucose metabolism.

Renal fibrosis is a key pathological feature of DKD, and PZP may regulate this process through multiple mechanisms. LRP2 belongs to the same family as LRP1 and is highly expressed in renal tubular epithelial cells. Sodium-glucose cotransporter 2 (SGLT2) inhibitors reduce LRP2 internalization, thereby ameliorating proximal tubular protein overload, mitochondrial morphological abnormalities, oxidative stress, and tubulointerstitial fibrosis (27). Transforming growth factor beta (TGF-β), a critical mediator contributing to renal fibrosis, reduces LRP2 expression, whereas this effect can be reversed by inhibiting the TGF-β type I receptor (TGF-βRI) (28). Therefore, we hypothesize that PZP may attenuate renal fibrosis by modulating the process of LRP2 internalization. In addition, PZP knockout activates the TGF-β/SMAD signaling pathway (a key pathway driving renal fibrosis) (29), suggesting another mechanism by which PZP influences renal fibrosis. PZP may also modulate inflammatory responses and redox signaling pathways to attenuate renal fibrosis, such as suppressing nuclear factor-kappa B (NF-κB) activity and activating antioxidant nuclear factor erythroid 2-related factor 2 (NRF2). Furthermore, PZP may inhibit aryl hydrocarbon receptor upregulation, which also contributes to the alleviation of renal fibrosis (30). Accumulating evidence indicates that DKD is associated with chronic inflammation and immune dysregulation. A recent study combining single-cell RNA sequencing and expression quantitative trait locus (eQTL) analysis identified PZP as a key gene involved in CKD and age-related renal dysfunction, primarily by regulating CD8 + central memory T cells. Consistently, GO and KEGG enrichment analyses in this study revealed that PZP may contribute to the occurrence and progression of DKD through immune- and complement-related pathways. In addition, PZP may be involved in CKD-associated vasculopathy by regulating nitric oxide (NO) bioavailability, inflammation, and protease activity (31).

In the present study, even after adjustment for confounding factors, multivariate logistic regression still demonstrated that plasma PZP concentrations were independently associated with DKD, although the effect size was relatively small (mild DKD: OR = 1.007; severe DKD: OR = 1.011). DKD is a chronic progressive disease induced by the accumulation of multiple pathogenic factors, and most biomarkers for such chronic progressive disorders usually exhibit small to moderate effect sizes. Although the effect size of PZP is modest, its association is stable and consistent, suggesting that PZP is more suitable for early risk stratification and long-term dynamic monitoring in diabetic patients rather than serving as a strong causal factor. Therefore, PZP can be used in combination with traditional renal function indicators, such as the eGFR and UACR, to improve the identification of high-risk populations with early or subclinical renal injury in diabetic patients, and it still carries important clinical significance. Public database analysis revealed that PZP expression correlated with reduced eGFR (*r* = −0.557, *p* = 0.011), increased serum Cr (*r* = 0.507, *p* = 0.019), and proteinuria (*r* = 0.761, *p* = 0.047) (22), confirming a potential link between PZP and renal function impairment, although the direction of this association and any causal relationship remain unclear.

Several limitations should be noted in this study. First, the cross-sectional design prevents us from determining whether elevated plasma PZP levels precede renal injury or result from renal damage, precluding the establishment of a causal link between plasma PZP levels and the development and progression of DKD. In addition, this study only demonstrated cross-sectional associations between plasma PZP levels and DKD-related parameters (such as eGFR, UACR, and metabolic indices) at a single time point, without evaluating dynamic changes in plasma PZP levels and renal function over time. Second, although major confounding factors were adjusted for in regression models, residual confounding from unmeasured factors (including medication use and duration of diabetes) cannot be completely excluded. Third, age distribution imbalances among the different groups (particularly in the validation cohort) may still represent a potential source of bias, even after adjustment for confounders. Fourth, the single-center design of this research limits the generalizability of the findings. Future multicenter studies with expanded sample sizes and more diverse populations are essential. Finally, although a correlation between plasma PZP levels and the UACR was observed in db/db mice, more robust animal and cell-based experiments are necessary to elucidate the precise role and underlying molecular mechanism of PZP in DKD.

## Conclusion

This study is the first to demonstrate that plasma PZP levels are independently correlated with DKD in both humans and mice and that PZP levels increase with DKD progression. PZP may have potential as a non-invasive biomarker for DKD patients. Further studies are needed to confirm its utility in predicting DKD onset, progression, and treatment response in the future.

## Data Availability

The raw data supporting the conclusions of this article will be made available by the authors, without undue reservation.

## References

[1] Afkarian M Zelnick LR Hall YN Heagerty PJ Tuttle K Weiss NS . Clinical manifestations of kidney disease among US adults with diabetes, 1988-2014. JAMA. (2016) 316:602–10. doi: 10.1001/jama.2016.10924 27532915 PMC5444809

[2] Zheng Y Ley SH Hu FB. Global aetiology and epidemiology of type 2 diabetes mellitus and its complications. Nat Rev Endocrinol. (2017) 14:88–98. doi: 10.1038/nrendo.2017.151, 29219149

[3] Federation, I.D. IDF Diabetes Atlas, Diabetes and Kidney Disease. Brussels: International Diabetes Federation (2023).

[4] Alicic RZ Rooney MT Tuttle KR. Diabetic kidney disease. Clin J Am Soc Nephrol. (2017) 12:2032–45. doi: 10.2215/CJN.11491116, 28522654 PMC5718284

[5] Sugahara M Pak WLW Tanaka T Tang SCW Nangaku M. Update on diagnosis, pathophysiology, and management of diabetic kidney disease. Nephrology. (2021) 26:491–500. doi: 10.1111/nep.13860, 33550672

[6] Selby NM Taal MW. An updated overview of diabetic nephropathy: diagnosis, prognosis, treatment goals and latest guidelines. Diabetes Obes Metab. (2020) 22:3–15. doi: 10.1111/dom.14007, 32267079

[7] Shora HA El-Deen IM El-Lithy NM Abo-Elmatty DM Khirallah SM. Growth differentiation factor-15: a marker for diabetic kidney disease in patients with metabolic-associated fatty liver disease. J Diabetes Complicat. (2025) 39. doi: 10.1016/j.jdiacomp.2025.109037, 40233467

[8] Qin L Zhang R Yang S Chen F Shi J. Knockdown of ANGPTL-4 inhibits inflammatory response and extracellular matrix accumulation in glomerular mesangial cells cultured under high glucose condition. Artif Cells Nanomed Biotechnol. (2019) 47:3368–73. doi: 10.1080/21691401.2019.1649274, 31387395

[9] Lin S Yu L Ni Y He L Weng X Lu X . Fibroblast growth factor 21 attenuates diabetes-induced renal fibrosis by negatively regulating TGF-β-p53-Smad2/3-mediated epithelial-to-mesenchymal transition via activation of AKT. Diabetes Metab J. (2020) 44:158. doi: 10.4093/dmj.2018.0235, 31701691 PMC7043973

[10] Wen X Zhou X Chen D Cheng J Ji L. Association between non-alcoholic fatty liver disease and diabetes-related microvascular complications: a retrospective cross-sectional study of hospitalized patients. Endocr Pract. (2022) 28:304–9. doi: 10.1016/j.eprac.2021.02.004, 33601024

[11] Yan Q Zhao Z Liu D Li J Pan S Duan J . Integrated analysis of potential gene crosstalk between non-alcoholic fatty liver disease and diabetic nephropathy. Front Endocrinol. (2022) 13:1032814. doi: 10.3389/fendo.2022.1032814, 36387855 PMC9642911

[12] Devriendt K Van den Berghe H Cassiman J-J Marynen P. Primary structure of pregnancy zone protein. Molecular cloning of a full-length PZP cDNA clone by the polymerase chain reaction. Biochimica et Biophysica Acta. (1991) 1088:95–103. doi: 10.1016/0167-4781(91)90157-H, 1989698

[13] Löb S Vattai A Kuhn C Schmoeckel E Mahner S Wöckel A . Spliceosome protein EFTUD2 is upregulated in the trophoblast of spontaneous miscarriage and hydatidiform mole. J Reprod Immunol. (2020) 140:103149. doi: 10.1016/j.jri.2020.103149, 32447180

[14] Tayade C Esadeg S Fang Y Croy BA. Functions of alpha 2 macroglobulins in pregnancy. Mol Cell Endocrinol. (2005) 245:60–6. doi: 10.1016/j.mce.2005.10.004 16297527

[15] Skornicka EL Kiyatkina N Weber MC Tykocinski ML Koo PH. Pregnancy zone protein is a carrier and modulator of placental protein-14 in T-cell growth and cytokine production. Cell Immunol. (2004) 232:144–56. doi: 10.1016/j.cellimm.2005.03.007, 15882859

[16] Wu Y Zhao Z Deng X Jia J Yuan G. Pregnancy zone protein, a potential research target in multiple diseases. Gene. (2025) 935:149013. doi: 10.1016/j.gene.2024.149013, 39433266

[17] Oliver-Williams C Johnson JD Vladutiu CJ. Maternal cardiovascular disease after pre-eclampsia and gestational hypertension: a narrative review. Am J Lifestyle Med. (2021) 17:8–17. doi: 10.1177/15598276211037964, 36636385 PMC9830232

[18] Fosheim IK Jacobsen DP Sugulle M Alnaes-Katjavivi P Fjeldstad HES Ueland T . Serum amyloid A1 and pregnancy zone protein in pregnancy complications and correlation with markers of placental dysfunction. Am J Obstet Gynecol MFM. (2023) 5:100794. doi: 10.1016/j.ajogmf.2022.100794, 36334725

[19] Rubio-Aliaga I Marvin-Guy LF Wang P Wagniere S Mansourian R Fuerholz A . Mechanisms of weight maintenance under high- and low-protein, low-glycaemic index diets. Mol Nutr Food Res. (2011) 55:1603–12. doi: 10.1002/mnfr.201100081, 21957032

[20] Jiang X Lin J Dong M Liu X Huang Y Zhang H . Overexpression of pregnancy zone protein in fat antagonizes diet-induced obesity under an intermittent fasting regime. Front Physiol. (2022) 13:950619. doi: 10.3389/fphys.2022.950619, 36051914 PMC9424687

[21] Lin J Jiang X Dong M Liu X Shen Q Huang Y . Hepatokine pregnancy zone protein governs the diet-induced thermogenesis through activating brown adipose tissue. Adv Sci. (2021) 8:e2101991. doi: 10.1002/advs.202101991, 34514733 PMC8564441

[22] Huang X Zhu C Lv S Wang Y Wang J Yuan S . Integrated single-cell transcriptome and eQTL analyses reveal the role of PZP in aging and chronic kidney disease. J Gene Med. (2025) 27:e70015. doi: 10.1002/jgm.70015 40058366

[23] Yong Q Huang C Chen B An J Zheng Y Zhao L . Gentiopicroside improves NASH and liver fibrosis by suppressing TLR4 and NLRP3 signaling pathways. Biomed Pharmacother. (2024) 177:116952. doi: 10.1016/j.biopha.2024.116952, 38917754

[24] Fedorenko A Lishko PV Kirichok Y. Mechanism of fatty-acid-dependent UCP1 uncoupling in brown fat mitochondria. Cell. (2012) 151:400–13. doi: 10.1016/j.cell.2012.09.010, 23063128 PMC3782081

[25] Chiabrando GA Vides MA Sánchez MC. Differential binding properties of human pregnancy zone protein– and α2-macroglobulin–proteinase complexes to low-density lipoprotein receptor-related protein. Arch Biochem Biophys. (2002) 398:73–8. doi: 10.1006/abbi.2001.2659, 11811950

[26] Chen J Zhang S Xue X Ma X Chen A Wu Y . Melatonin attenuates kidney injury by alleviating lysosomal damage in diabetic kidney disease. Acta Biochim Biophys Sin. (2025) 57:1589–600. doi: 10.3724/abbs.2025034, 40509901 PMC12616729

[27] Otomo H Nara M Kato S Shimizu T Suganuma Y Sato T . Sodium-glucose cotransporter 2 inhibition attenuates protein overload in renal proximal tubule via suppression of megalin O-GlcNacylation in progressive diabetic nephropathy. Metabolism. (2020) 113:154405. doi: 10.1016/j.metabol.2020.154405, 33069809

[28] Ahmad A Cabezas F Farfán P Marzolo M-P. Participation of the SMAD2/3 signalling pathway in the down regulation of megalin/LRP2 by transforming growth factor beta (TGF-ß1). PLoS One. (2019) 14:e0213127. doi: 10.1371/journal.pone.0213127 31120873 PMC6532859

[29] Vrljicak P Myburgh D Ryan AK van Rooijen MA Mummery CL Gupta IR. Smad expression during kidney development. Am J Physiol Renal Physiol. (2004) 286:F625–33. doi: 10.1152/ajprenal.00152.2003, 14656760

[30] Wang M Hu H-H Chen Y-Y Chen L Wu X-Q Zhao Y-Y. Novel poricoic acids attenuate renal fibrosis through regulating redox signalling and aryl hydrocarbon receptor activation. Phytomedicine. (2020) 79:153323. doi: 10.1016/j.phymed.2020.153323, 32920287

[31] Chen W-L Liao W-T Hsu C-N Tain Y-L. Pregnancy zone protein as an emerging biomarker for cardiovascular risk in pediatric chronic kidney disease. J Clin Med. (2023) 12:5894. doi: 10.3390/jcm12185894, 37762835 PMC10531502

